# Promotion of Resilience and Emotional Self-Care in Families and Health Professionals in Times of COVID-19

**DOI:** 10.3389/fpsyg.2022.879846

**Published:** 2022-03-29

**Authors:** Óscar Sánchez-Hernández, Merav Barkavi-Shani, Rosa María Bermejo

**Affiliations:** ^1^Department of Evolutionary and Educational Psychology, University of Murcia, Murcia, Spain; ^2^Department of Personality, Evaluation and Psychological Treatment, University of Murcia, Murcia, Spain

**Keywords:** self-care, resilience, well-being, prevention, families, health professionals, pandemic, COVID-19

## Introduction

Populations affected by the COVID-19 pandemic have a significantly higher prevalence of depression, anxiety, insomnia, post-traumatic stress disorder, and psychological stress compared to the general population under normal circumstances, as indicated by the meta-analysis by Cénat et al. (2021). Other studies with large samples carried out in different countries indicate that throughout the confinement, there is a progressive worsening of mental health (Ammar et al., 2020). On the other hand, we know that the application and increase in the levels of social distancing have been necessary, since the results of the studies find that they have conduced to a significant reduction in the spread of infection and the number of deaths (Daghriri and Ozmen, 2021). Some studies indicate that the population may be hard to adhere to these restrictions and the population may have fatigue in performing social distancing rules (Harvey, 2020; Shirali et al., 2021).

Gilbody et al. (2021) note that the mental health research community has been successful in describing the nature of the impact of COVID-19, but less so in generating solutions and providing clinical trial data to establish what works to mitigate the impacts. We suggest employing interventions to promote resilience in families. We also reflect on the need for the emotional self-care of mental health professionals in this context of a pandemic.

## Promotion of Resilience in Families in the Face of Confinement in the Covid-19 Pandemic

A study by Zayas et al. (2021) points to the need for interventions to promote resilience for the general population in times of pandemic, alluding to the study on the preliminary analyzes of the “Resilience and Well-being Programme: Stay at Home” (Sánchez-Hernández and Canales, 2020a,b), which is a psycho-educational intervention applied in confinement and de-escalation of the first wave of the pandemic in Spain, to promote well-being and prevent emotional problems (see Table 1). The sample consisted of 259 participants, 68.3% women and 31.7% men, with a mean age of 39.33 years (SD = 14.3). The group that received the Resilience and Well-being Programme was made up of 80 participants and the control group by 179 (Sánchez-Hernández and Canales, 2021). Participants reported high satisfaction with the programme and improvement, from pretest to post-test, in resilience, psychological well-being, post-traumatic stress symptoms, ease of handling the pandemic, and mood. The analyzes of differences between the groups indicate, in a statistically significant way, that the group to which the programme was applied has fewer symptoms of post-traumatic stress, greater ease of dealin g with the pandemic, and better mood, compared to the control group in the post-test (Sánchez-Hernández, 2020b; Sánchez-Hernández and Canales, 2021). The analyzes (Sánchez-Hernández, 2020a; Sánchez-Hernández and Canales, 2021) indicate that satisfaction with the programme, following th e programme's guidelines, and the development of certain programme skills, predict following prudent and healthy behaviors during confinement (e.g., staying home, wearing masks, social distancing, hand washing, and follow sanitary recommendations in general). These results are encouraging and have yet to be confirmed with experimental design studies.

**Table 1:** Well-being Pills from the resilience and wellness program: “stay at home” (Sánchez-Hernández and Canales, 2020b).

**Well-being Pills**	**Main theoretical approaches**	**References**	**Main applied content**
1. Resilience	Psychoeducation Motivational Interview Positive Psychology	Forés and Grané, 2008; Sánchez-Hernández et al., 2009	Accept and understand that it is normal to have doubts. Find out from reliable sources (professionals and health centers...) and follow their recommendations. Doing so is a sign of social altruism. Choose some time of the day to update the information avoiding unnecessary excess information. Identify and reflect on the helpful factors that promote personal and family resilience.
2. Family Strengths	Behavioral Activation and Positive Psychology	Peterson and Seligman, 2004; Sánchez-Hernández et al., 2019	Keep doing pleasant activities and move your body (sports, dance, listen to music...) establishing a schedule to do them. Keep socializing, following the health recommendations. Identify and use personal and family psychological strengths. Take care of your diet, healthy sleep habits and sunbathe from your terrace, balcony or window.
3. Emotional management	Emotional Education	Sánchez-Hernández et al., 2019	Accept and understand all your emotions (and those of others), both negative and positive. Regulate your emotions with deep breathing, positive imagery, powerful postures, calming self-talk, mindfulness, and sharing them with trusted people.
4. Love and Secure Attachment	Positive Psychology, attachment, and Cognitive-Behavioral Therapy	Seligman et al., 2005; Hoffman et al., 2019;	Make time to play and connect with your loved ones. Kindly welcome and embrace the emotions of your loved ones. Encourage constructive communication with your family and friends.
5. Hope	Positive Psychology and Acceptance and Commitment Therapy	Snyder, 2000; Ciarrochi et al., 2016	Reflect on strengths, supports, skills and abilities that you have used in challenging situations in the past, which will help generate hope. Be grateful and appreciate what you have and what happens in your day to day.
6. Optimism	Positive Psychology and Cognitive Therapy	Seligman et al., 2005; Sánchez-Hernández et al., 2016	The naive optimist denies reality and tends to recklessness. The exaggerated pessimist becomes blocked and makes others uncomfortable. The intelligent optimist sees reality and has a plan of action to deal with it. You can schedule limited time for excessive worrying and debating catastrophic thoughts.
7. Life Sense	Positive Psychology	Steger, 2009	How can we improve as a society from this experience? In the future, when all this happens… How would you like to relate to your grandchildren the attitude with which you faced all this? What values would be important to you at this time?

## Emotional Self-Care of Mental Health Professionals

Mental Health professionals are at increased risk of burnout, due to the emotional demands of their work. Studies found that the prevalence of burnout among them can range from 20 to 40 percent (O'Connor et al., 2018; Laverdière et al., 2019; Yang and Hayes, 2020). In particular, during the COVID-19 pandemic, health professionals have been at heightened risk of depression, anxiety, burnout, insomnia and other disorders (Morin and Carrier, 2020; Al-Humadi et al., 2021; Olashore et al., 2021; Rajabimajd et al., 2021). The COVID-19 pandemic has caused heavy psychological impact among healthcare professionals especially women and frontline workers (Sun et al., 2021). In addition to this, healthcare providers may receive public stigma (Patel et al., 2021).

Positive psychology interventions (PPI) are intentional activities that aim to cultivate positive feelings, behaviors, or cognitions (Seligman et al., 2004). In a review of published papers on 15 PPIs conducted at places of work, in the period 2000–2011, improvements had been found, after interventions, in happiness, positive mood, positive emotions, vigor, positive self-perception, psychological capital (PsyCap-hope, optimism, and self-efficacy) and resilience, and reductions in stress, depression, and anxiety (Meyers et al., 2013).

Previous interventions have focused mostly on strengthening self-compassion, as a way helping professionals to cope with stress and prevent burnout, through mindfulness training or loving kindness meditation (i.e., Boellinghaus et al., 2014). A type of intervention found to be beneficial in work settings has been positive psychology. One of the basic models of such interventions is the PERMA model for well-being, designed by Peterson et al. (2005) and Seligman (2002, 2011). A fundamental exercise in positive psychology would be the counting of daily blessings (gratitude). In a gratitude intervention, participants are encouraged to reflect on, and acknowledge, things they are grateful for.

Mead et al. (2021) examined protective factors during the first wave of lockdown on the well-being in of 138 participants from the United Kingdom and found that “gratitude” and “tragic optimism” were identified as key positive psychological attributes contributing to well-being.

Lakioti et al. (2020) presented a study to investigate the factors that help therapists maintain their resilience to work stressors. Participants were Greek mental health practitioners (*N* = 163). The study suggests that positive psychology concepts are useful variables in the mental health professionals' resilience field. Particularly meaning, positive emotions, and satisfaction with relationships might play an important role in the development of strategies for improving therapists' mental health and functioning. Litam (2021) conducted a study used a national sample of professional counselors (*N* = 161) providing services during the COVID-19 pandemic to examine the extent to which perceived stress, coping response, resilience, and posttraumatic stress predict burnout, secondary traumatic stress, and compassion satisfaction. The results indicated that resilience had a strong positive relationship with compassion satisfaction and a strong negative relationship with burnout. Perceived stress was also strongly positively related to burnout. This study's findings emphasize the importance for professional counselors to cultivate resilience and self-care practices during the pandemic, and perhaps during crisis and disaster mental health counseling more generally. This finding has led to daily gratitude exercises being recommended for everyone, and but specifically for mental health professionals; it would be beneficial for them to write three good things that happened to them each day. It was found in meta- analyzes that the most effective interventions at work were: gratitude and strengths (Donaldson et al., 2019). Moreover, the positive impact of gratitude has been found to contribute to longevity, well-being, and a reduction in psychopathology (Jans-Beken et al., 2020).

## Discussion

The meta-analysis by Cénat et al. (2021) indicates that since the COVID-19 pandemic has broken out, the population-wide prevalence of depression, anxiety, insomnia, post-traumatic stress disorder, and psychological stress has risen markedly. In Greece (Lakioti et al., 2020) found that positive psychology concepts are useful variables in the mental health professionals' resilience to work stressors.

As the article by Gilbody et al. (2021) points out, the mental health research community has been successful in describing the nature of the impact of COVID-19, but less so in generating solutions and providing clinical trial data to establish what works to mitigate the effects of COVID-19 impacts. It is necessary to analyze this imbalance and promote clinical trial studies to promote well-being, prevent emotional problems, and carry out early detection and intervention of emotional disorders. We suggest employing interventions to promote resilience in families (Sánchez-Hernández et al., 2022). We also reflect on the need for the emotional self-care of mental health professionals in the context of a pandemic through the daily practice of gratitude exercises involving “three good things that happened to me.”

## Author Contributions

ÓS-H carried out mainly the section Promotion of Resilience in Families in the Face of Confinement in the COVID-19 pandemic. MB-S and RB mainly carried out the section Emotional Self-Care Of Mental Health Professionals. The rest of the sections have been carried out by the three authors. All authors contributed to the article and approved the submitted version.

## Conflict of Interest

The authors declare that the research was conducted in the absence of any commercial or financial relationships that could be construed as a potential conflict of interest.

## Publisher's Note

All claims expressed in this article are solely those of the authors and do not necessarily represent those of their affiliated organizations, or those of the publisher, the editors and the reviewers. Any product that may be evaluated in this article, or claim that may be made by its manufacturer, is not guaranteed or endorsed by the publisher.
